# Linkers Having a Crucial Role in Antibody–Drug Conjugates

**DOI:** 10.3390/ijms17040561

**Published:** 2016-04-14

**Authors:** Jun Lu, Feng Jiang, Aiping Lu, Ge Zhang

**Affiliations:** 1 Institute for Advancing Translational Medicine in Bone & Joint Diseases, School of Chinese Medicine, Hong Kong Baptist University, Hong Kong, China; ljaaa111@163.com (J.L.); jiangfenghz@163.com (F.J.); 2 Institute of Integrated Bioinfomedicine & Translational Science, Hong Kong Baptist University Shenzhen Research Institute and Continuing Education, Shenzhen 518000, China; 3 Institute of Precision Medicine and Innovative Drug Discovery, HKBU (Haimen) Institute of Science and Technology, Haimen 226100, China

**Keywords:** antibody–drug conjugates, cytotoxic drug, monoclonal antibody, linker, attachment site, tumor

## Abstract

Antibody–drug conjugates (ADCs) comprised of a desirable monoclonal antibody, an active cytotoxic drug and an appropriate linker are considered to be an innovative therapeutic approach for targeted treatment of various types of tumors and cancers, enhancing the therapeutic parameter of the cytotoxic drug and reducing the possibility of systemic cytotoxicity. An appropriate linker between the antibody and the cytotoxic drug provides a specific bridge, and thus helps the antibody to selectively deliver the cytotoxic drug to tumor cells and accurately releases the cytotoxic drug at tumor sites. In addition to conjugation, the linkers maintain ADCs’ stability during the preparation and storage stages of the ADCs and during the systemic circulation period. The design of linkers for ADCs is a challenge in terms of extracellular stability and intracellular release, and intracellular circumstances, such as the acid environment, the reducing environment and cathepsin, are considered as the catalysts to activate the triggers for initiating the cleavage of ADCs. This review discusses the linkers used in the clinical and marketing stages for ADCs and details the fracture modes of the linkers for the further development of ADCs.

## 1. Introduction

Antitumor drug development has made great progress since the 20th century, especially with the emergence of biological products, promoting better selectively for the different kinds of antitumor drugs. However, traditional chemotherapy drugs with strong cytotoxicity face long-standing problems in terms of lacking specificity and targeting effects, which kill the tumor cells, accidentally injure normal cells and cause serious adverse reactions [1,2]. The monoclonal antibodies appear to possess very specific targeting tumor cells, whereas there are limited treatment effects for solid tumors due to their large molecular weight causing poor penetrability.

**Figure 1:**
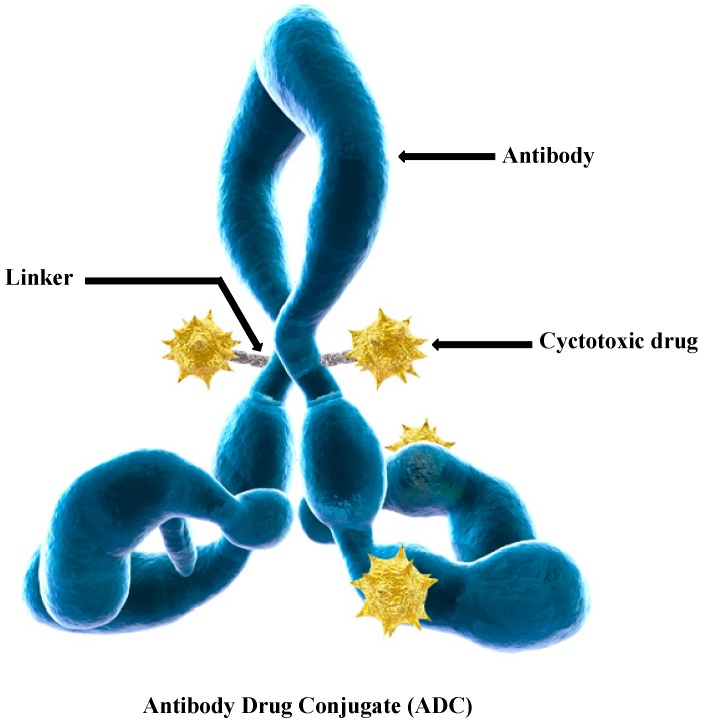
Schematic for the structure of an antibody–drug conjugate (ADC). Adapted from reference [6].

In recent years, tumor targeted therapy has been a research hot spot on account of their good targeting properties and potent anti-tumor activities. Antibody–drug conjugates (ADCs) have made significant progress in tumor therapy and show a promising future. ADCs as a novel class of highly potent biopharmaceutical drugs conjugate a cytotoxic drug with a monoclonal antibody (mAb) through an applicable linker (Figure 1). ADCs take advantage of the highly active cell-killing of cytotoxic molecules and their superior binding specificity; meanwhile, they prolong the half-life of cytotoxic molecules or decrease their dose-limiting toxicity [3,4,5].

**Figure 2:**
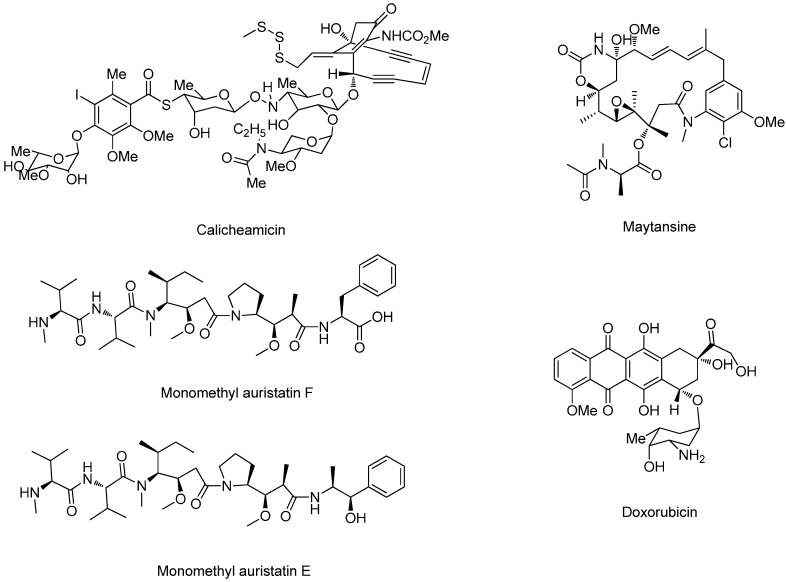
The structural formula of calicheamicin, maytansine, monomethyl auristatin F (MMAF), monomethyl auristatin E (MMAE), doxorubicin.

The cytotoxic drugs used for conjugating antibodies must meet three requirements: strong cell toxicity, possessing the appropriate modified site from where the conjugate releases the original drug in the tumor cell, and a definite action mechanism. However, the cytotoxic drugs chosen for effective traditional chemotherapy drugs that were prepared for the early ADCs, and were subjected to preclinical and clinical tests, showed low biological activity. For instance, BR96-Doxorubicin (DOX) was found to be less effective than its free cytotoxic drug [7]. Similarly, another conjugate named KSI/4-DAVLBHYD also showed lower activity than the parent compound [8]. Since then, a series of small molecular compounds were discovered or synthesized with higher cytotoxicity and better sensitivity to different tumor cells than the traditional chemotherapy drugs, such as calicheamicin, doxorubicin, auristatins and maytansine (Figure 2).

The highly potent cytotoxic drugs were divided into two main categories: microtubulin-disrupting drugs and DNA-modifying drugs. Those drugs were prepared for targeting conjugates, which entered various stages of clinical trials and demonstrated hundreds to thousands times higher potency compared to the traditional chemotherapy drugs (Table 1) [9].

Antibodies that selectively bind to the tumor cells and show little cross-reactivity with healthy tissues can be conjugated to the specific cytotoxic drugs through different linkers [30,31]. Antibodies with a preferential role in ADCs must have the following characteristics: special targeting ability to deliver cytotoxic drugs to the tumor cells, high affinity binding to tumor cell-surface antigens and the ability to induce the cells’ endocytosis, low immunogenicity and the appropriate linkage sites which would not impact the affinity, endocytosis or efficacy of cytotoxic drugs [32]. However, recently, one report indicated low binding affinity to antibodies may allow for effective penetration to solid tumors [33]. The binding affinity ability of antibody-antigens is significant for appropriate delivery of cytotoxic drugs and internalization into the tumor cells [34]. Antibody engineering involving the optimization and designation of antibody frames promotes humanized antibodies transformed from animal-derived antibodies and reduces the potential risks of immunogenicity while providing therapeutic antibodies [35].

Considerable efforts are being invested to construct the appropriate linkers, which must meet requirements for maintaining the properties of monoclonal antibodies and the cell killing activity of cytotoxic drugs, reducing systemic toxicity, maintaining the stability of ADCs and releasing in the right circumstances [4,5,36]. Among these properties, linkers should possess two crucial characteristics, including stability in plasma for an extended period of time so that the ADCs can reach and localize to the cancer cell in the original formation. After internalization, the linkers play a role as a trigger for releasing the cytotoxic drugs when the ADCs face particular circumstances in the cancer cells, and the released cytotoxic drugs could bind to their targets [37]. With the particular microenvironment of tumor cells and the delivery mechanism selected, the active formation of the cytotoxic drug may be efficiently released from ADCs by fracturing the designed linkers inside the target cells [38]. The stability and rupturing capacity of linkers affect the overall pharmacokinetics (PK) properties, toxicities and therapeutic indexes of ADCs [39]. The earlier ADCs, such as BR96-DOX and Mylotarg@, did not have a sufficient therapeutic index and had been withdrawn from the market, which was attributed to the poor stability of linkers [4,40].

## 2. Drug Release Strategies for Antibody–Drug Conjugates (ADCs)

**Figure 3:**
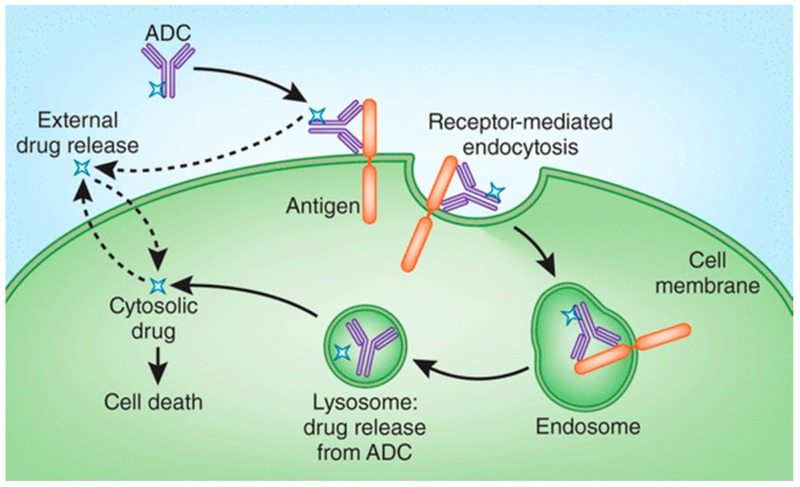
Schematic representation of the mechanism of drug delivery mediated by ADCs. Reproduced with permission from reference [41]. Solid line arrows indicate specific tumor cell killing through receptor-mediated endocytosis. Dash line arrows elicit the specific tumor cell killing through extracellular drug release.

The primary advantages of ADCs are that they can act as prodrugs during systemic circulation and finally release the free drugs at the target tumor cells. The operation mechanism of successful ADCs is depicted as below (Figure 3). Relying on highly targeted tumor antigen recognition and effective internalization, ADCs recognize and bind to a specific tumor antigen on the cell surface, then internalization of ADCs is conducted through endocytosis. Once entering the tumor cells, the ADCs are transferred to the endosome or lysosome which digest the potential linkers or antibodies and actively release cytotoxic drugs. Therefore, linkers play a crucial role in releasing the potent drug at target tumor cells.

Linkers are classified according to different categories in terms of the mechanism of drug release and their stability in circulation, including cleavable linkers and non-cleavable linkers [32,42]. Cleavable linkers rely on the physiological environment, such as there being high glutathione concentrations, low pH, and special protease, which could assist the linkers in enabling chemical or biochemical reactions by way of hydrolyzation or proteolysis [3,43]. Non-cleavable linkers despond on the monoclonal antibody degradation after ADCs’ internalization within the lysosomes and endosomes to generate the metabolites containing the active cytotoxic drugs with or without a portion of the linkers. On account of different mechanism strategies, differences between the potential ADC metabolisms and the varying characteristics of cytotoxic drugs should be taken into consideration [44].

Each release strategy must account for many factors: the various activities of cytotoxic drugs, the characteristics of monoclonal antibodies, and the particular disease. The optimal linkers designed to conjugate the cytotoxic drugs to monoclonal antibodies must meet the particular requirements imposed by the factors described above.

## 3. Types of Linkers

The two main parts of linkers, including the non-cleavable linkers and the cleavable linkers, play profound roles in determining the pharmacokinetic properties, therapeutic index, selectivity and the overall success of the ADC. With the development of ADCs introduced to clinical trials or approved by the FDA, a series of typical linkers have been exploited [31].

### 3.1. Non-Cleavable Linkers

Many non-cleavable linkers have been explored in ADC development. The greatest advantage of non-cleavable linkers compared to cleavable linkers is their increased plasma stability. Non-cleavable linked ADCs have outperformed their cleavable counterparts *in vivo* in several studies, and mAb degradation within the lysosome after ADC internalization is required for non-cleavable linkers to release active drug [45]. Non-cleavable linkers can potentially provide a greater therapeutic window compared to cleavable linkers, due to the fact that the payload derivative from non-cleavable ADCs can kill the target cells [43,46]. In addition, a potentially reduced off-target toxicity compared to the cleavable linker conjugates is expectable, as non-cleavable ADCs can provide greater stability and tolerability.

**Figure 4:**
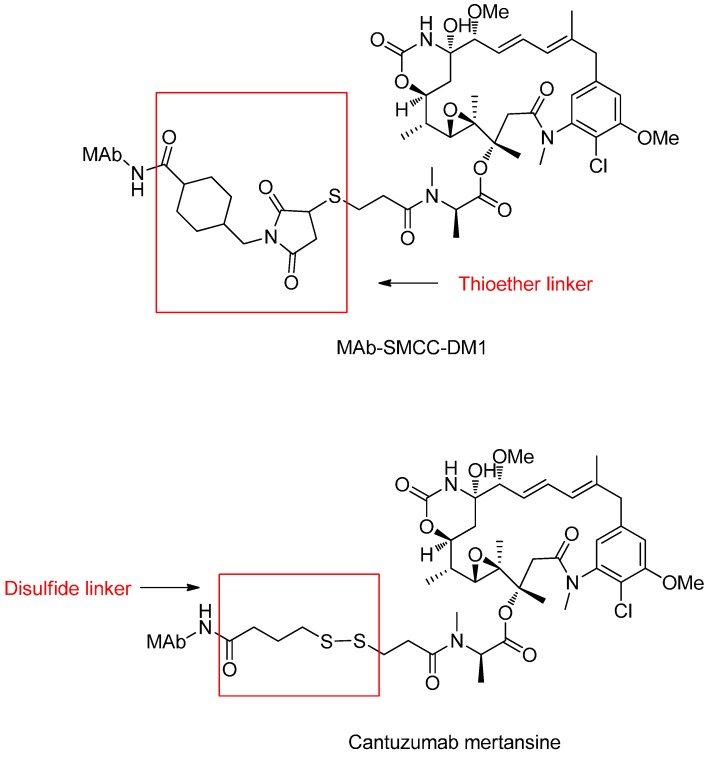
The structural formula of huC242-SMCC-DM1 and cantuzumab mertansine. Adapted from reference [47].

Yelena *et al.* synthesized the huC242-SMCC-DM1 conjugate binding DM1 to the humanized monoclonal antibody (huC242) via an *N*-succinimidyl-4-(*N*-maleimidomethyl)cyclohexane-1-carboxylate (SMCC) for non-cleavable thioether linker and tested the cytotoxicity of conjugate on COLO 205 cells and Namalwa cells. Compared with the cantuzumab mertansine (huC242-DM1) containing a cleavable disulfide linker, the huC242-SMCC-DM1 is efficacious only against tumors in which all proliferating cells express the target antigen, and displayed significantly lower *in vivo* activity in multiple xenograft tumor models (Figure 4) [47].

**Figure 5:**
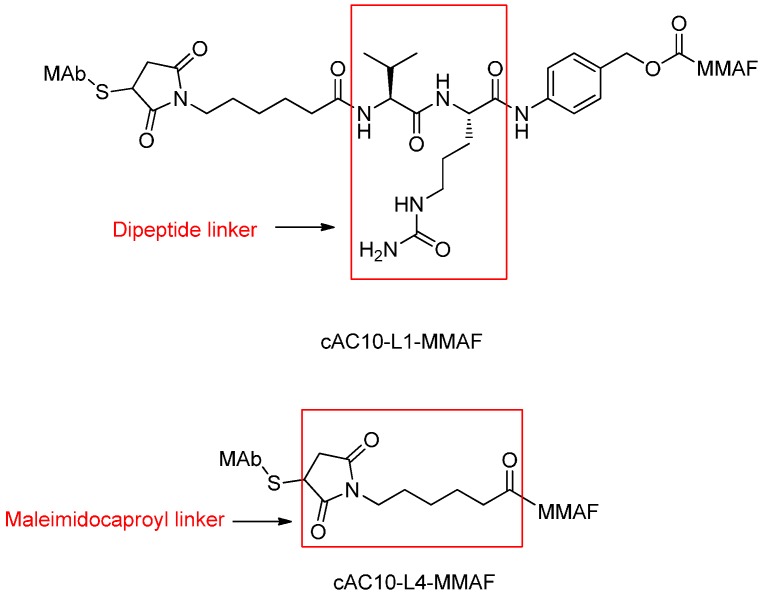
The structural formula of cAC10-L1-MMAF and cAC10-L4-MMAF. Adapted from reference [48,49].

The cAC10-L4-MMAF in which cAC10 (anti-CD30) linked to the antimitotic auristatin derivative MMAF via a non-cleavable maleimidocaproyl linker was approximately as potent as cAC10-L1-MMAF with a dipeptide linker *in vitro* against a large panel of cell lines and was equally potent *in vivo* (Figure 5) [48,49].

**Figure 6:**
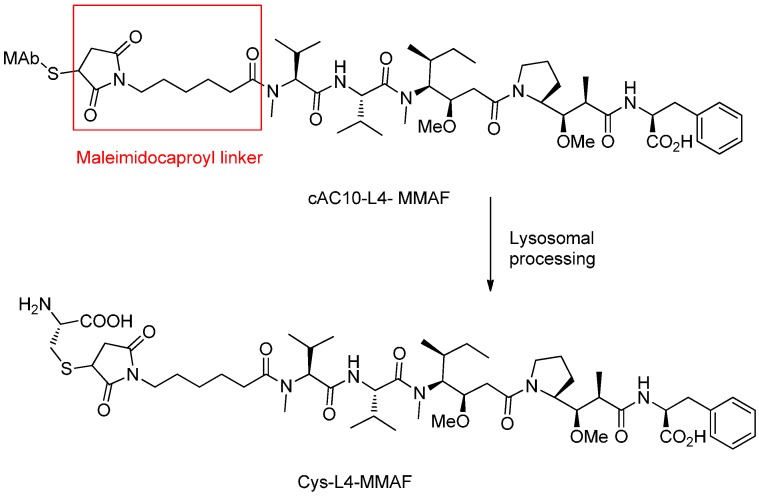
The structural formula of the cAC10-L4-MMAFand the supposed cleavage mechanism after internalization into the lysosome. Adapted from reference [43,50].

The drug released from cAC10-L4-MMAF was the cysteine-L4-MMAF adduct analyzed by LCMS, which likely arises from monoclonal antibody degradation within the lysosome of targeted cells (Figure 6) [43].

In the same way, a humanized anti-CD70 mAb was conjugated to the anti-microtubule agent MMAF via the non-cleavable maleimidocaproyl linker and formed another ADC SGN-75. In the clinical trial, SGN-75 inhibited the growth of human carcinomas and improved potency *in vitro* by increasing the drug-loading, without substantial effects on the PK properties and pharmacodynamic (PD) *in vivo* [49,51].

### 3.2. Cleavable Linkers

The cleavable linkers play a crucial role in the success of an ADC, being stable in the blood circulation for a long period of time and efficiently being released in the tumor microenvironment, for both the chemically labile linkers and enzyme cleavable linkers.

#### 3.2.1. Chemically Labile Linkers

The chemically labile linkers, including acid-cleavable linkers and reducible linkers, are extensively applied to the ADCs since they are able to undergo fracture, increasing the acidity of the endosomal–lysosomal pathway and the concentration of glutathione inside cells.

##### Acid-Cleavable Linkers

Acid-cleavable linkers, such as hydrazone, are specifically designed to remain stable at the neutral pH of blood circulation, but undergo hydrolysis and release the cytotoxic drug in the acidic environment of the cellular compartments. These linkers have been associated with non-specific release of the drug in clinical studies [4].

**Figure 7:**
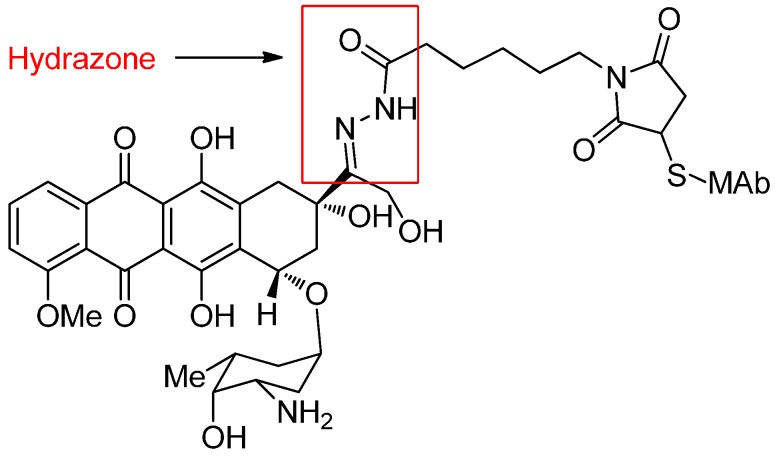
The structural formula of BR96-doxorubicin. Adapted from reference [52].

The BR96-Doxorubicin (BR96-Dox) as an excellent example is constructed by conjugating doxorubicin to the monoclonal antibody BR96 through an acid-cleavable hydrazone (Figure 7). After reaching and binding to the target tumor cells, BR96-Dox is internalized through the endocytosis into lysosomes [52]. In clinical trials, BR96-Dox has been found to not be associated with the typical side-effect profile of native doxorubicin and could potentially deliver high doses of doxorubicin to antigen-expressing tumors, which has been found to enable complete remission and cure subcutaneous human breast, lung and colon tumors [53,54].

**Figure 8:**
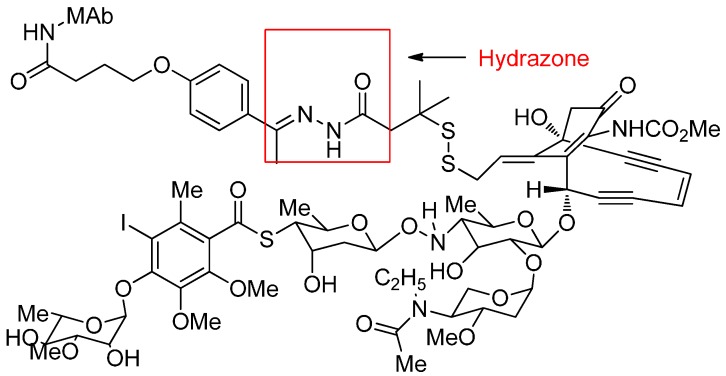
The structural formula of BR96-doxorubicin. Adapted from reference [56].

Mylotarg, withdrawn from the US market in 2010, was the first approved ADC for treatment of CD33-positive acute myeloid leukaemia. This ADC consists of a semisynthetic derivative of calicheamicin and a recombinant monoclonal antibody (hP67.6) directed against the CD33 antigen through an acid-cleavable hydrazone (Figure 8) [55].

However, the weakness of Mylotarg is likely due to the insufficiently stable chemical linker, which relies on a pH-dependent release mechanism, and too many of the drugs are being released in the bloodstream [57]. Nonetheless, CMC-544 (inotuzumab ozogamicin), targeted to CD22 expressed by B-lymphoid malignancies and covalently conjugated to calicheamicin through an acid-labile 4-(4′-acetylphenoxy) butanoic acid linker, shows good stability both in human plasma and serum, the structure of which is closely related to Mylotarg [58,59,60]. IMMU-110, being evaluated in a Phase I/II study, is comprised of doxorubicin (DOX) linked to the humanized anti-CD74 monoclonal antibody via an acid-liable hydrazone, which showed high activity against MM, and appeared to be safe in a monkey model of MM cells [61,62]. In terms of development of DOX-based ADCs, IMMU-115 with a hydrazone linker provides the basis for novel therapeutic approaches to B-cell malignancies [63].

##### Reducible Linkers

Reducible linkers take advantage of the difference in reduction potential in the intracellular compartment *versus* plasma. Reduced glutathione presented in tumor cells’ cytoplasma is up to 1000-fold higher than that present in normal cells’ cytoplasma, and the tumor cells also contain enzymes of the protein disulfide isomerase family, which may contribute to reduction of the disulfide bond in cellular compartments [64,65]. The linkers of disulfide bonds keep conjugates intact during systemic circulation, and are selectively cleaved by the high intracellular concentration of glutathione, releasing the active drugs at the tumor sites from the non-toxic prodrugs [66].

**Figure 9:**
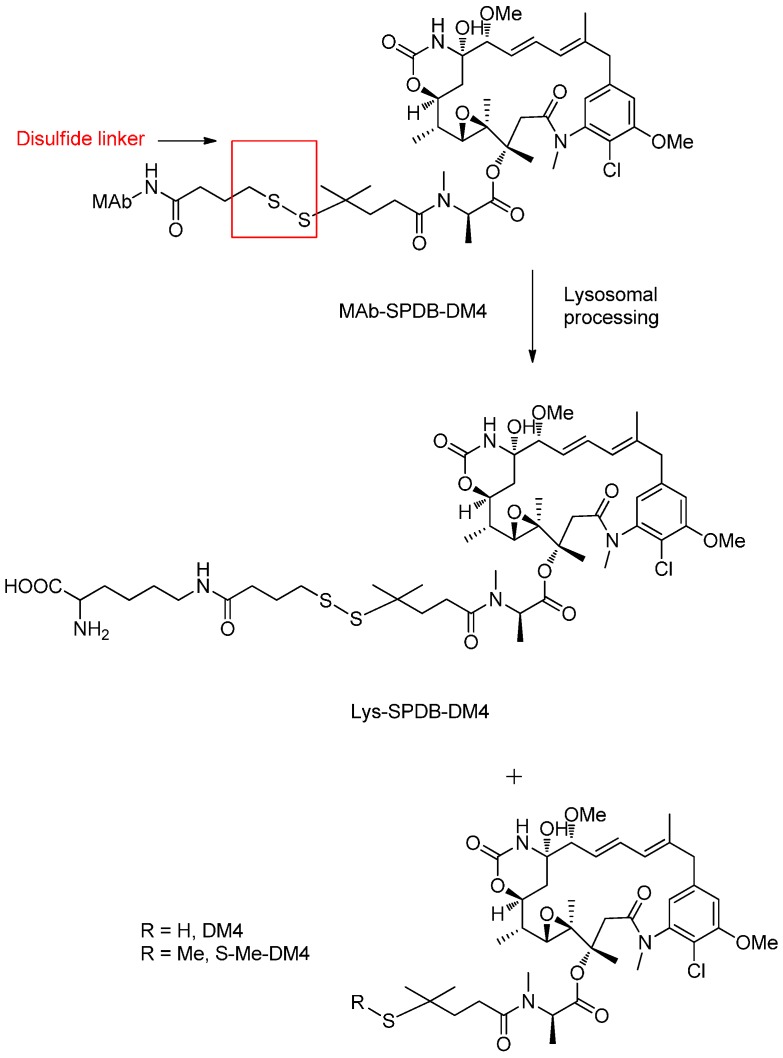
The structural formula of huC242-SPDB-DM4 and the supposed cleavage mechanism after internalization into the lysosome. Adapted from reference [49,67].

Representative disulfide linker-based conjugates contain the cytotoxic maytansinoids conjugated to the different monoclonal antibodies. In particular, huC242-SPDB-DM4 (IMGN242) is a novel ADC comprised of huC242 antibody conjugated to the potent maytansinoid via the cleavable disulfide-linker, which allows targeted delivery to pancreatic tumor cells and releases the potent maytansinoid in tumor cells (Figure 9) [67,68].

Compared with uncleavable huC242-SMCC-DM1 containing a thioether linker, huC242-SPDB-DM4 with an average of three to four maytansinoid molecules showed the approximate activities *in vitro* [69]. However, huC242-SPDB-DM4 exhibited significantly higher activity in multiple xenograft tumor models *in vivo*. The conjugate, which was linked via a disulfide bond exert an excellent effect and clearance rate for the conjugate, was about four times faster than that for the antibody component [47].

**Figure 10:**
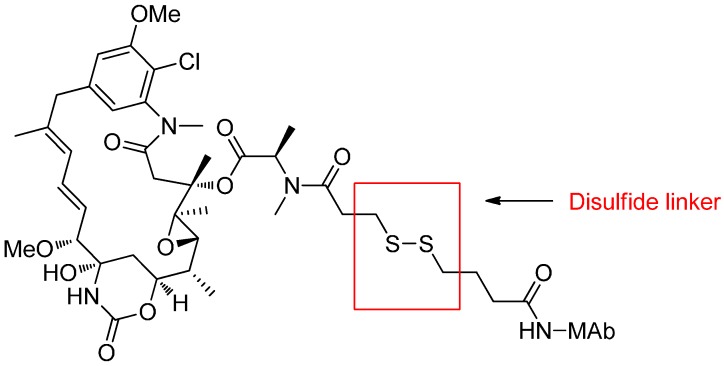
The structural formula ofIMGN901. Adapted from reference [72].

IMGN901 consists of a potent maytansinoid attached to a CD56-binding monoclonal antibody through a disulfide linker in a Phase II clinical trial, which is a novel CD56-targeting anticancer agent and expressed on virtually all Merkel Cell Carcinoma (MCC) tumors (Figure 10) [70,71].

#### 3.2.2. Enzyme Cleavable Linkers

Unlike the chemically labile linkers discussed above, enzyme cleavable linkers take advantage of the abundance of hydrolytic enzymes with the specificity to recognize the sequences of peptides or patterns of carbohydrate in order to degrade peptides and carbohydrates. The different contents of these enzymes between the blood and lysosomal compartment ensure a well-designed ADC undergoes cleavage only in the lysosomal environment.

##### Peptide-Based Linkers

The peptide-based linkers are designed to keep ADCs intact in systemic circulation, and allow easy release of the cytotoxic drugs upon cleavage by specific intracellular proteases, such as cathepsin B [73]. Due to unsuitable pH conditions and serum protease inhibitors, these peptide linkers show greater systemic stability with rapid enzymatic release of the drug in the target cell, such as valine-citrulline (Val-Cit) dipeptide linker, phenylalanine-lysine (Phe-Lys) dipeptide linker. This linker has been utilized in many ADCs in the clinic, which displays an excellent balance between plasma stability and intracellular protease cleavage [74].

**Figure 11:**
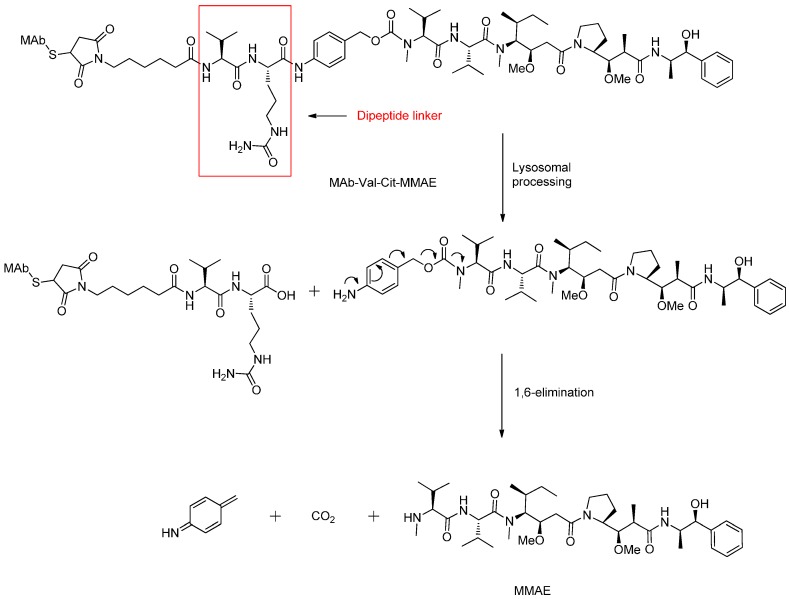
The structural formula of MAb-Val-Cit-MMAE (SGN-35) and the supposed cleavage mechanism after internalization into the lysosome. Adapted from reference [49,76].

In order to enhance the antitumor activity of CD30-directed therapy, the cytotoxic drug monomethyl auristatin E (MMAE) was conjugated to a CD30-specific monoclonal antibody via a protease-cleavable dipeptide linker forming the ADC brentuximab vedotin (SGN-35) [41]. It displayed good tolerability and antitumor activity for the CD30^+^ hematologic malignancies in a clinical study (Figure 11) [75].

Similarly, AGS-5ME consists of the anti-tubule drug MMAE and the anti-AG5-5ME mAb composed by a XenoMouse-derived fully human IgG_2_k monoclonal antibody, via a Val-Cit dipeptide linker. After attaching to the cell surface, the AGS-5ME is internalized and releases the free cytotoxic drug by the proteolytic cleavage. At present, the AGS-5ME is in a Phase I clinical trial for the treatment of pancreatic cancer and prostate cancer [77,78].

##### β-Glucuronide Linker

Another type of enzyme-labile linker is β-glucuronide linker exploited in ADC and the cytotoxic drug undergoes release and cleavage by β-glucuronidase, an enzyme present in lysosomes or the tumor interstitium abundantly presenting in lysosomes, and is overexpressed in some tumors [79,80,81]. Having hydrophilic properties, this linker could reduce the ADC aggregation of the hydrophobic drugs and promote the solubility of the intact ADC compared to the dipeptide-based ADC [82].

**Figure 12:**
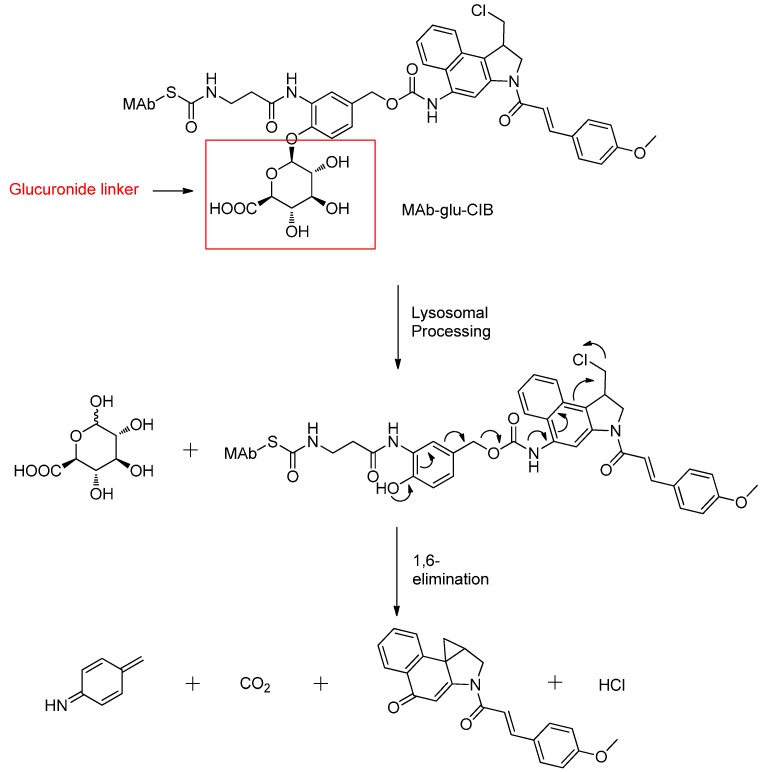
The structural formula of MAb-glu-CIB and the supposed cleavage mechanism after internalization into the lysosome. Adapted from reference [83].

Jeffrey reported the antibody–drug conjugates linking MAbs cAC10 (anti-CD30) and h1F6 (anti-CD70) to cyclopropyl indole minor-groove binders (MGBs) via a β-glucuronide linker. The β-glucuronide moiety does not directly link to the ADC with the payload, however, cleavage by β-glucuronidase could trigger 1,6-elimination of the spacer liberating the free cytotoxic drug (Figure 12). The water-soluble β-glucuronide linker is stable in plasma, effectively delivers drugs to target cells and allows for potent activities comparable to that of a free cytotoxic drug [79].

**Figure 13:**
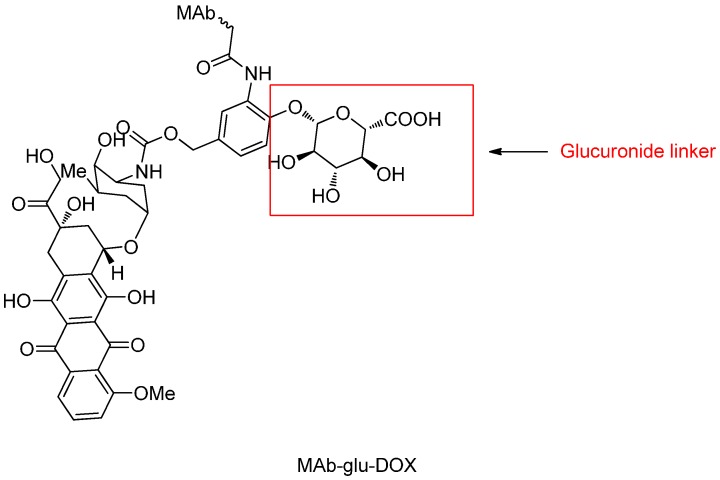
The structural formula of MAb-glu-DOX. Adapted from reference [79].

The β-glucuronide linker has been utilized to conjugate multiple monoclonal antibodies in a series of ADCs to deliver different cytotoxic drugs including auristatin derivatives MMAE, MMAF and doxorubicin propyloxazolin (Figure 13) [84].

The ADCs showed high levels of immunologically specific cytotoxic activity on cancer cell lines, respectively. From the trial results, the β-glucuronide linker system shows the effective strategy for targeting cytotoxic drug and provides ADCs with high degrees of efficacy at well-tolerated doses [85].

The first ADC linker derived from acid-labile hydrazones was designed to be cleaved inside target cancer cells, but inevitably underwent premature spontaneous release of the drug, which caused damage to normal tissues. The next linkers were disulfides bond and enzyme-labile linkers that have achieved greater stability *in vivo*. To decrease the damage from payload to non-target tissues, noncleavable linkers were recently developed. However, cytotoxic payloads must accommodate substitutions while maintain the potency. Linkers can be modified to be appropriate for different modes of metabolism or activation. For instance, conjugates containing peptide linkers or disulfide linkers may allow a faster rate of activation and release of the cytotoxic drugs than ADCs with non-cleavable linkers, which necessitate cleavage of two bonds at both N- and C-termini of the amino acid of attachment. Some cytotoxic payloads are good substrates for the development of noncleavable linkers on account of accommodating substitutions and maintaining the potency, however, other cytotoxic payloads which can’t tolerate substitutions require a cleavable linker.

## 4. Attachment Sites on the Antibodies for Linkers

The attachment sites on the antibodies are important considerations for design and assessment of ADCs, which could be attribute in large effects to the chemical groups on linkers [86]. From the clinical trials of ADCs, application of available lysines or reduced cysteine disulfides to form the conjugates is the predominant approach. The lysine and cysteine as the natural amino acids exist in the antibodies with different contents and are treated with diverse methods to prepare ADCs, including heterogeneous ADCs and homogeneous ADCs [87]. The heterogeneous ADCs were generally synthesized by utilizing the thiol groups from reduction of cystines and primary amino group of lysines directly. However, the heterogeneity of ADCs resulted in pharmacokinetic limitations. Comparing with heterogeneous ADCs, homogeneous ADCs through antibody engineering and other techniques to provide the specific sites are more stable and have better activities *in vivo* [86,88,89].

**Figure 14:**
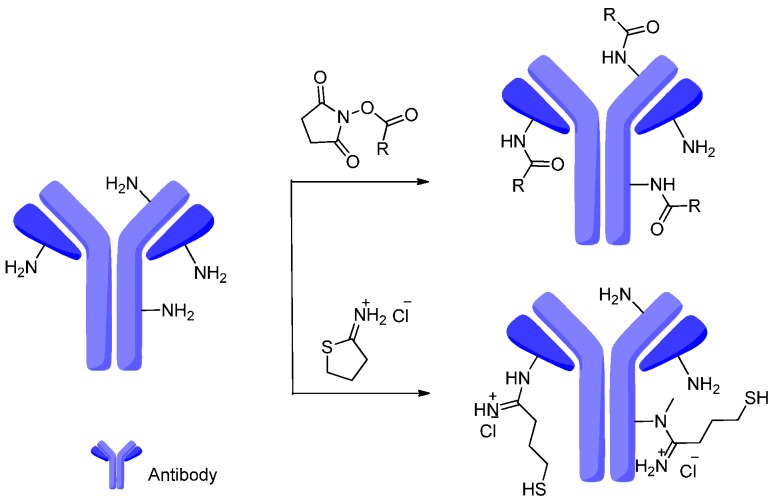
The amines of lysines on the antibody react with *N*-hydroxysuccinimide (NHS) esters forming the amides and react with Traut’s reagent forming the amidines.

Generally, lysines with free amines are more prevalent than cysteines with disulfides and are less uniformly distributed in the antibody. The primary amine in the lysines easily reacted with *N*-hydroxysuccinimide (NHS) esters incorporated into the drug-linker to form stable amide and a great number of commercial linkers depend on this method (Figure 14) [55,90]. Meanwhile, the amine of lysine also was applied to make an amidine with a pendant thiol for connection to a linker or payload via 2-imiothiolane (Traut’s reagent).

**Figure 15:**
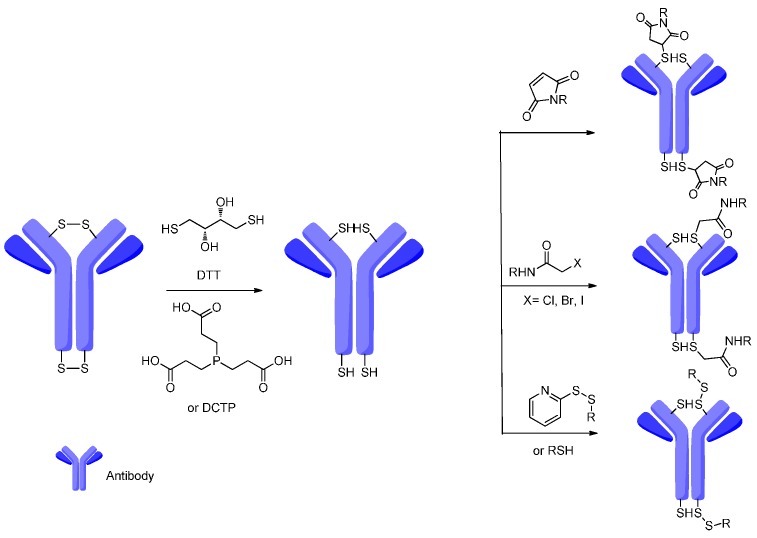
The disulfides of cysteines on the antibody were reduced by DL-Dithiothreitol (DTT) or Tris(2-carboxyethyl)phosphine (TCEP) and the thiols react with maleimide, halogenoalkane, disulfide or thiol compounds.

Cysteines as natural amino acids in the antibodies are tethered through disulfide bridges, whereas reducing the disulfide bonds should rarely affect functions of a monoclonal antibody [91]. Under carefully controlled conditions, the interst and disulfide bonds could be selectively reduced by the DL-Dithiothreitol (DTT) or Tris(2-carboxyethyl)phosphine (TCEP) and provide reactive thiol groups; meanwhile, intrachain disulfide bonds maintain their original state. The free thiol groups as attachment sites on the antibodies can be conjugated with a small linker molecule through different chemical reactions, such as Michael additions, a-halo carbonyl alkylations and disulfide formation (Figure 15) [92,93,94].

**Figure 16:**
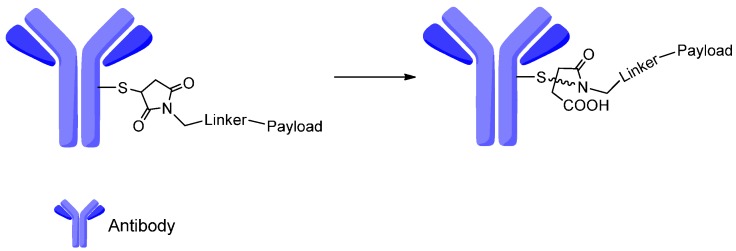
The hydrolysis of the succinimide-thioether ring results in a “ring-opened” linker. Adapted from reference [95].

However, the maleimide-based ADCs were reported to be prone to losing payload through the retro–Michael reaction with existence of blood thiols, particularly albumin [95,96]. The hydrolysis of the succinimide-thioether rings in the ADCs is a promising method to avoid the retro–Michael reaction occuring (Figure 16). The ADCs containing the hydrolyzed succinimide-thioether linker displayed improved stability, PK exposure and efficacy as compared to the non-hydrolyzed analogs [97].

The attachment sites on the antibodies employing the natural amino acids do not require preliminary modifications and allow for efficient reactions to take place. However, the disadvantages of the non-specific attachment sites could lead to variability and heterogeneity among conjugates. The heterogeneous conjugates containing diverse drugs are difficult to purify and characterize, which might influence ADC PK and stability [98,99].

With the development and requirement of homogeneous ADCs, site-specific antibodies have been obtained via the technology of interchain cysteine cross-linking, besides the recombinant approaches [100]. In recent years, some novel cysteine-reactive functionalities have been developed to yield site-specific antibody fragments or full antibodies via the insertion of specially designed chemical molecules or groups into native disulfide bonds, such as pyridazinedione, dibromopyridazinedione, dibromomaleimide, and bis-alkylating bis-sulfone groups [101,102,103]. Compared to analogous heterogeneous ADCs, the homogeneous ADCs prepared from site-specific antibodies exhibit reduced toxicity and superior efficacy *in vivo* [104,105].

As a consequence, significant efforts have been invested to explore diverse sites and develop homogeneous conjugates. The antibodies with site-specific chemoselectivity not only minimize the possibility of conjugate heterogeneity, but also have the potential to decrease payload–linker interference with antibody–receptor recognition [44]. Insertion of an unnatural amino acid with a bio-orthogonal reactive handle, enzymatic conjugation and insertion or mutation of cysteine residues in the antibody sequence are the main three strategies [39].

**Figure 17:**
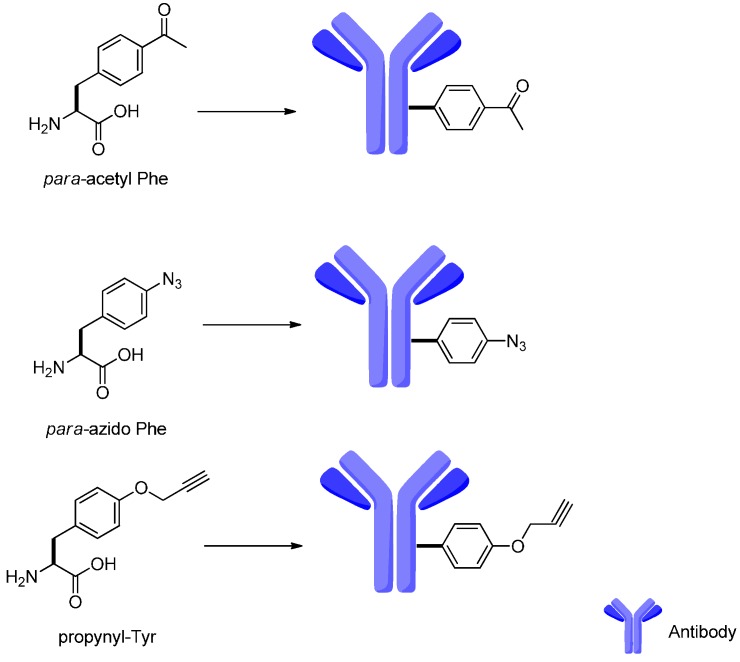
The commonly employed unnatural amino acids in the antibodies: *para*-acetyl Phe, *para*-azido Phe and propynyl-Tyr.

The challenge for the next generation of ADCs is the generation of antibodies with genetically encoded unnatural amino acids. Although attempts at the introduction of more than 30 unnatural amino acids to antibodies have been made, only three of them with chemical handles were found to have high value and were applied (Figure 17) [106,107,108].

**Figure 18:**
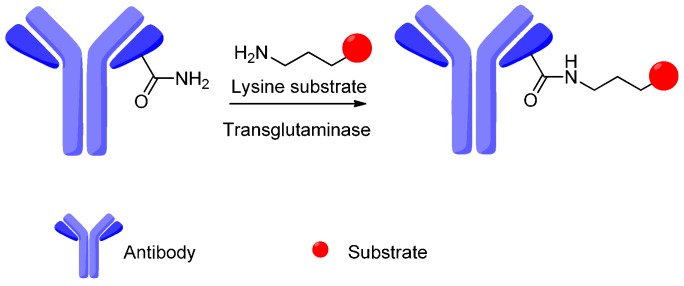
A glutamine side chain is ligated to a lysine side chain by transglutaminase.

A novel bioorganic conjugation approach for preparing the site-specific labeling of proteins was reported recently, which utilized enzymatic post-translational modification processes. Jegar *et al.* reported that bacterial transglutaminase catalyzed the primary amine of lysine residues’ ligation with glutamine side chains (Figure 18) [109].

Both attachment sites on the antibodies and chemical groups on the linkers not only determine the conjugation efficiency and production feasibility of ADCs, but also affect the stability and integrity of the conjugates during duction and storage as well as during clinical treatment.

## 5. Conclusions

The non-cleavable linkers, hydrazone linkers, disulfide linkers, peptide linkers and β-glucuronide linkers are most frequently utilized in the ADCs. Significant efforts have been made in designing and choosing suitable linkers for conjugating monoclonal antibodies and cytotoxic drugs. Those linkers can influence the stability, toxicity, PK properties, and pharmacodynamics of ADCs. Each linker has its advantages and disadvantages, and many factors must be considered when they are selected and applied for determinate monoclonal antibodies and specific cytotoxic drugs. The appropriate linker must consider the existing groups presented in the monoclonal antibody, the reactive groups in the cytotoxic drugs, as well as the derivative functional groups. The perfect linker can guarantee sufficient stability of cytotoxic drugs during circulation in the blood stream, effectively prevent premature drug release, efficiently facilitate the liberation of the cytotoxic drug at the target tumor cells, and vigorously promote the efficacy and tolerability of successful ADCs.

## Figures and Tables

**Table 1:** List of antibody–drug conjugates (ADCs) in clinical trials and Food and Drug Administration (FDA)-approved.

ADC	Antibody/Liker	Cytotoxic Drug	Tumor Type	Side Effects	Status	Reference
Gemtuzumab ozogamicin	Humanized IgG4, hP67/6 Hydrazone	Calicheamicin	Acute Myeloid Leukemia (AML)	Myelo-suppression	Withdrawn	[10]
Inotuzumab ozogamicin	Humanized IgG4, G5/44 Hydrazone	Calicheamicin	B-cell lymphomas	Nausea, fever	Phase II/III	[11]
Immunomedics (IMMU)-110 (hLL1-DOX)	Milatuzumab Hydrazone	Doxorubicin	Multiple myeloma	Not specified	Phase I/II	[12]
Lorvotuzumab mertansine (IMGN901)	Humanized IgG1, huC242 Disulfide	Maytansinoid	Multiple myeloma, solid tumors	Headache, fatigue	Phase I/II	[13]
IMGN242 (huC242-DM4)	Humanized IgG1, huC242 Disulfide	Maytansinoid	Solid tumors	Corneal deposits, keratitis	Phase II	[14]
SAR566658	Humanized IgG1, DS6 Disulfide	Maytansinoid	Solid tumors	Keratitis	Phase I	[15]
BT-062	Anti-CD138 chimeric IgG4 Disulfide	Maytansinoid	Multiple myeloma	Mucositis, stomatitis	Phase I/II	[16]
BAY 94–9343	Anti-mesothelin fully human IgG1 Disulfide	Maytansinoid	Mesothelin-positive solid tumours	Not specified	Phase I	[17]
IMGN388	Anti-integrin, IgG1 Disulfide	Maytansinoid	Solid tumors	Headache, confusion	Phase I	[18]
BIIB015	Anti-cripto IgG1 Disulfide	Maytansinoid	Anti-cripto, solid tumors	Neuropathies	Phase I	[19]
SAR3419 (huB4-DM4)	huB4, humanized IgG1 Disulfide	Maytansinoid	B-cell Non-Hodgkin’s lymphoma	Peripheral neuropathies	Phase II	[20]
Brentuximab vedotin (SGN-35)	Anti-CD30 Dipeptide	Auristatin	Lymphomas	Nausea, fatigue	Approved	[21]
Glembatumumab vedotin CDX-011	Anti-CR011 Dipeptide	Auristatin	Breast cancer	Rash, alopecia	Phase I/II	[22]
SGN-75	Anti-CD70 Dipeptide	Auristatin	Renal cell carcinoma	Fatigue, nausea	Phase I	[23]
AGS-22M6E	Anti-Nectin fully human IgG Dipeptide	Auristatin	Solid tumours	Not specified	Phase I	[24]
PSMA ADC	Anti-PSMA fully human IgG1 Dipeptide	Auristatin	Metastatic, hor-mone-refractory prostate cancer	Not specified	Phase I	[25]
Trastuzumab-DM1 (T-DM1)	Trastuzumab, humanized IgG1 Thioether	Maytansinoid	Metastatic breast cancer	Photophobia, conjunctivitis	Phase III	[26]
Brentuximab vedotin (T-DM1)	ChIgG1 Thioether	Maytansinoid	Breast cancer	Nausea, headache	Approved	[27]
IMGN529	K7153A humanized IgG1 Thioether	Maytansinoid	B cell malignancies	Nausea	Phase I	[28]
AMG595	Anti-EGFRvIII Fully human IgG1 Thioether	Maytansinoid	Glioblastoma	Not specified	Phase I	[29]
